# Rasmussen Aneurysm and Fungal Co-infection in a Healthy Young Adult With Tuberculosis

**DOI:** 10.7759/cureus.89186

**Published:** 2025-07-31

**Authors:** Huria Huma, Doaa Subahi, Monica Rajendran, Nawazish Karim

**Affiliations:** 1 Respiratory Medicine, University Hospitals of Leicester NHS Trust, Leicester, GBR; 2 Cardiology, University Hospitals of Leicester NHS Trust, Leicester, GBR

**Keywords:** active pulmonary tuberculosis, cavitating pneumonia, pulmonary fungal infection, rasmussen’s aneurysm, staphylococcal pneumonia

## Abstract

A 24-year-old British Indian male experienced a severe and complex course of cavitating pneumonia caused by a rare co-infection with *Staphylococcus aureus*, *Mycobacterium tuberculosis*, and a non-*albicans Candida *species. He initially presented with symptoms of community-acquired pneumonia and was treated with antibiotics and subsequently discharged. Four days later, he re-presented with hemoptysis, hypoxia, and sepsis, requiring intensive care admission. Imaging revealed extensive cavitating lesions in the right lower lobe, empyema, pneumothorax, and a Rasmussen aneurysm. Management included 24 h in the intensive care unit, multiple chest drains, embolization of the aneurysm, and a three-month course of combined antibiotic, antifungal, and antituberculous therapy. Comprehensive immunological workup, including HIV testing, was negative, confirming the patient’s immunocompetent status. This case highlights the extreme rarity of such a multifaceted pulmonary co-infection in a young, otherwise healthy individual, and underscores the importance of early identification and aggressive management of concurrent infections and rare but life-threatening complications such as Rasmussen aneurysm and invasive fungal co-infection.

## Introduction

Cavitating pneumonia can result from a variety of pathogens, including *Staphylococcus aureus*, anaerobes, and *Mycobacterium tuberculosis*. While uncommon in community-acquired pneumonia, lung cavities have occasionally been reported with organisms such as *Streptococcus pneumoniae *and *Haemophilus influenzae *[1]. Among these, tuberculosis (TB) presents unique diagnostic challenges due to its insidious onset, broad clinical spectrum, and potential to mimic more typical bacterial infections.

Tuberculosis-related complications can be extensive, particularly in the presence of co-infections such as respiratory fungal diseases. One rare but potentially life-threatening complication is a Rasmussen aneurysm, which is a pseudoaneurysm of a pulmonary artery that develops adjacent to a tuberculous cavity due to progressive arterial wall erosion. It typically presents with hemoptysis and occurs most often in middle-aged or older individuals with chronic, cavitary pulmonary TB, especially in the setting of immunosuppression or delayed diagnosis. In a review of 174 reported cases, men were affected more than twice as often as women; however, the rupture rate was notably higher in female patients [2].

This case is particularly notable for its presentation in a young, immunocompetent male with no significant past medical history, which is atypical for Rasmussen aneurysm. It underscores the diagnostic complexity and clinical risk posed by concurrent bacterial and mycobacterial infections, which led to severe cavitary lung disease and vascular complications. This report highlights the importance of early recognition and coordinated multidisciplinary care in managing rare but serious respiratory presentations, even in patients without classic risk factors.

## Case presentation

**Figure 1:**
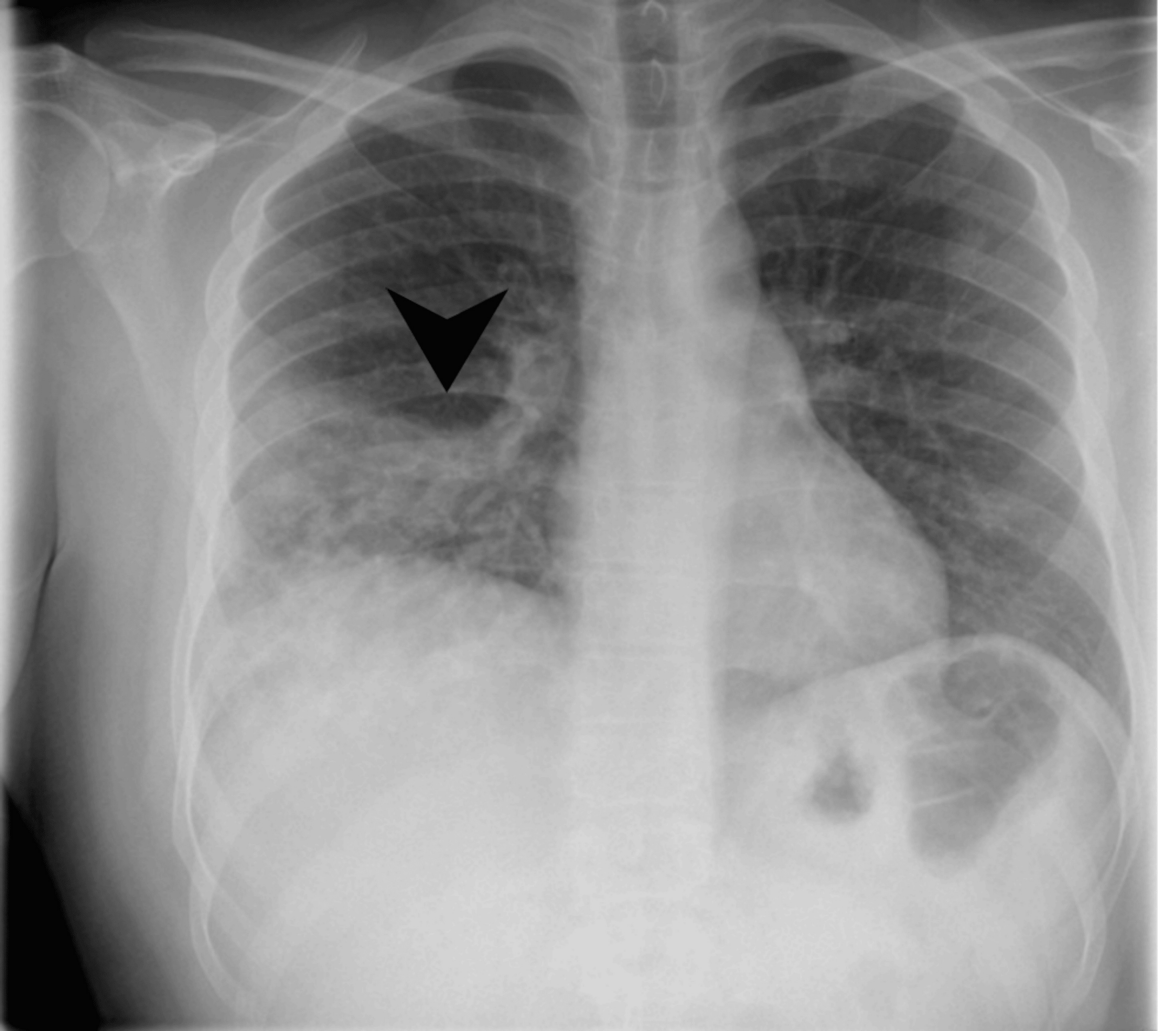
Posteroanterior chest radiograph showing right lower lobe consolidation (arrowhead) with a focal area of increased opacity in the right lower lung zone.

A 24-year-old British Indian male presented to the hospital in May 2023 with a one-month history of productive cough and a five-day history of fever, headache, chest tightness, and coryza. At the time of the initial presentation, the patient was clinically stable, with a blood pressure of 147/77 mmHg, heart rate of 91 bpm, temperature of 36.9°C, and oxygen saturation of 98% on room air. On examination, he was borderline febrile at 37.3°C, with a heart rate of 100 bpm. Chest auscultation revealed right-sided crepitations and reduced air entry, findings that raised concern for a localized lower respiratory tract infection. A chest X-ray showed right basal consolidation, consistent with pneumonia (Figure 1). The markedly elevated C-reactive protein at 182 mg/L further supported an acute inflammatory process. Based on these findings and the absence of any acute distress or underlying chronic lung disease, the assessing doctor determined that outpatient management was appropriate. Given the typical presentation, a diagnosis of community-acquired pneumonia was made. He was treated with 24 h of intravenous co-amoxiclav, followed by four days of oral co-amoxiclav, and discharged the following day with a five-day course of oral co-amoxiclav.

Given the patient’s stable condition and lack of red flag features at the time, further investigations such as sputum cultures, serological workup, or chest computed tomography (CT) were not deemed necessary during the initial encounter. Follow-up was arranged with the pneumonia nursing team, including a repeat chest X-ray scheduled for six weeks after discharge to monitor radiological resolution and ensure appropriate recovery.

**Figure 2:**
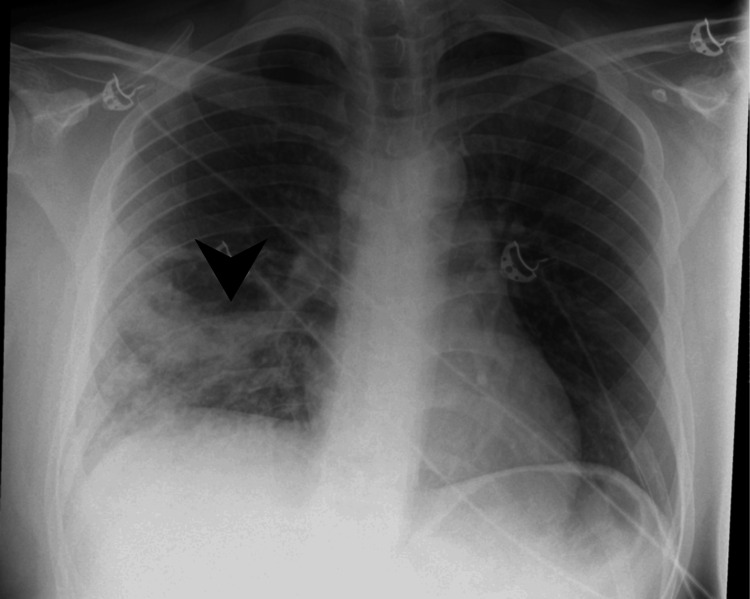
Follow-up posteroanterior chest radiograph showing progression of right lower lobe consolidation (arrowhead). In comparison to the previous image, there is an increase in the extent and density of the right lower lung opacity.

Four days following discharge, the patient re-presented with hemoptysis and worsening shortness of breath. On assessment, he was hemodynamically unstable, with a blood pressure of 87/60 mmHg, a heart rate of 154 bpm, and a temperature of 39.3°C. Inflammatory markers had significantly worsened, and arterial blood gas analysis on 15 L oxygen revealed persistent hypoxemia with oxygen saturation of 90%. A chest X-ray at re-presentation demonstrated worsening consolidation (Figure 2). A sputum sample with a mucopurulent appearance was collected for culture, which grew *Staphylococcus aureus*. Additionally, urinary screening for atypical pneumonia pathogens was negative. Key laboratory findings at this time are summarized in Table 1.

**Table 1:** Summary of key laboratory and arterial blood gas results on re-presentation. WCC: white cell count; CRP: C-reactive protein

Test	Result	Reference range
WCC	13.3 × 10⁹/L	4.0-11.0 × 10⁹/L
CRP	249 mg/L	<5 mg/L
Neutrophil count	10.69 × 10⁹/L	1.50-7.50 × 10⁹/L
PaO_2_	9.65 kPa	10.5-13.5 kPa
PaCO_2_	4.06 kPa	4.7-6.0 kPa
K	3.3 mmol/L	3.5-5.0 mmol/L
Na	129 mmol/L	135-145 mmol/L
Lactate	1 mmol/L	0.5-2.2 mmol/L

**Figure 3:**
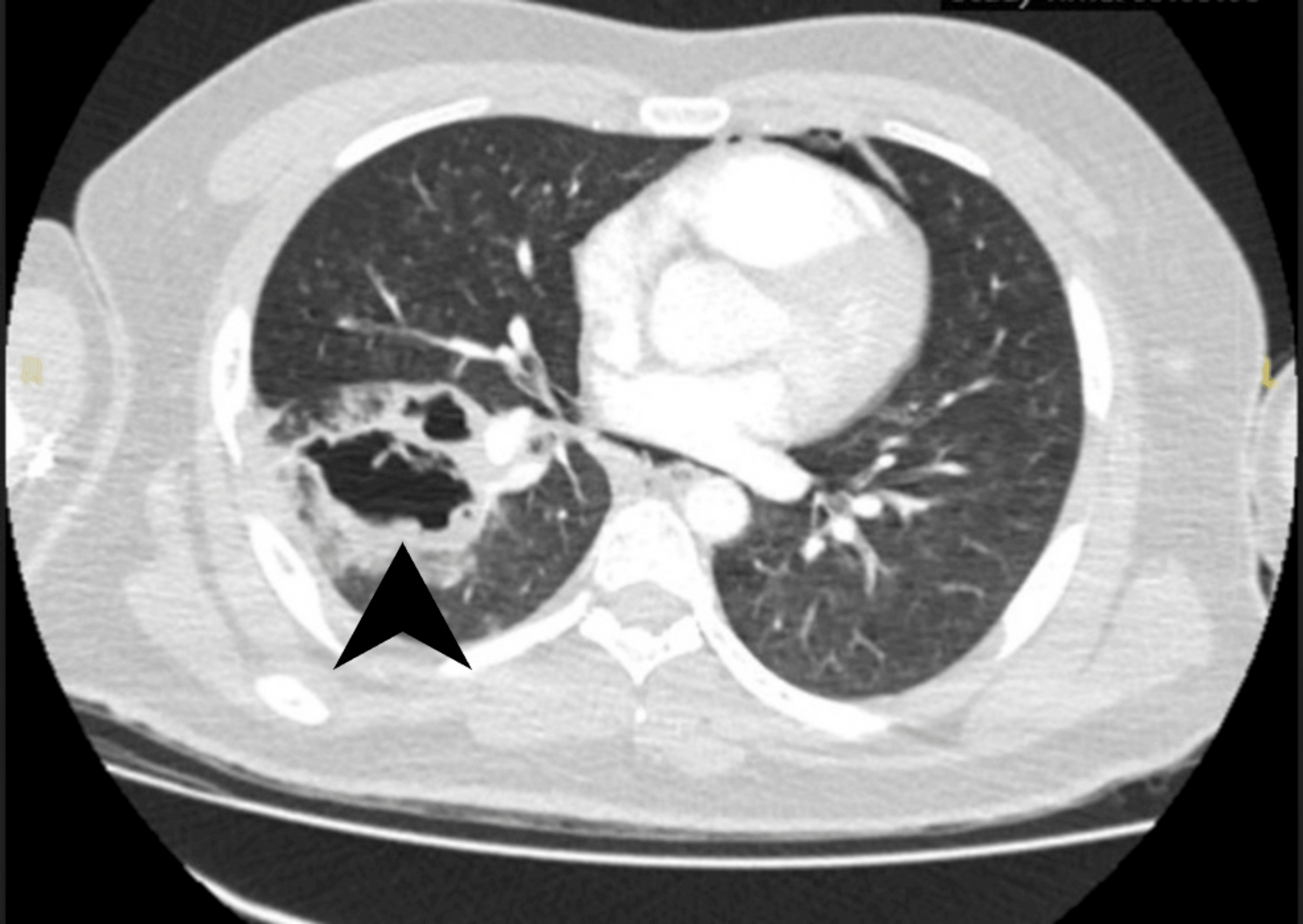
Axial contrast-enhanced CT of the thorax showing cavitating pneumonia in the right lower lobe with associated pneumomediastinum. Arrowhead points to the cavitary lesion, with surrounding features of pneumomediastinum. CT: computed tomography

Empirical treatment with intravenous meropenem and oral clarithromycin was initiated, and the patient was transferred to the intensive care unit (ICU) with suspected cavitating pneumonia and sepsis. On his second day in the ICU, the TB PCR result from a sputum sample collected on the day of readmission - before CT pulmonary angiography (CTPA) - returned positive; results were available 48 h later. This was a critical finding, given the patient’s triad of recurrent hemoptysis, cavitating lesions on imaging, and systemic inflammatory signs, features strongly suggestive of pulmonary tuberculosis. The CTPA, performed later on the day of readmission, confirmed extensive right lower lobe cavitations, tree-in-bud nodularity (a radiological hallmark of endobronchial spread of infection), moderate empyema, and pneumomediastinum, consistent with cavitating tuberculosis (Figure 3).

These findings underscore the importance of early suspicion for TB, even in immunocompetent individuals presenting with atypical respiratory infections. Notably, the patient was born and raised in the United Kingdom, with no history of travel to TB-endemic regions, no known exposure to tuberculosis, no prior TB screening, and no underlying conditions suggesting increased susceptibility.

He was commenced on standard quadruple antituberculous therapy (isoniazid, rifampicin, ethambutol, and pyrazinamide). High-flow oxygen support was initially required; later, weaned to 2 L/min of oxygen via nasal cannula, and the patient was stepped down to the infectious diseases unit (IDU) after three days.

**Figure 4:**
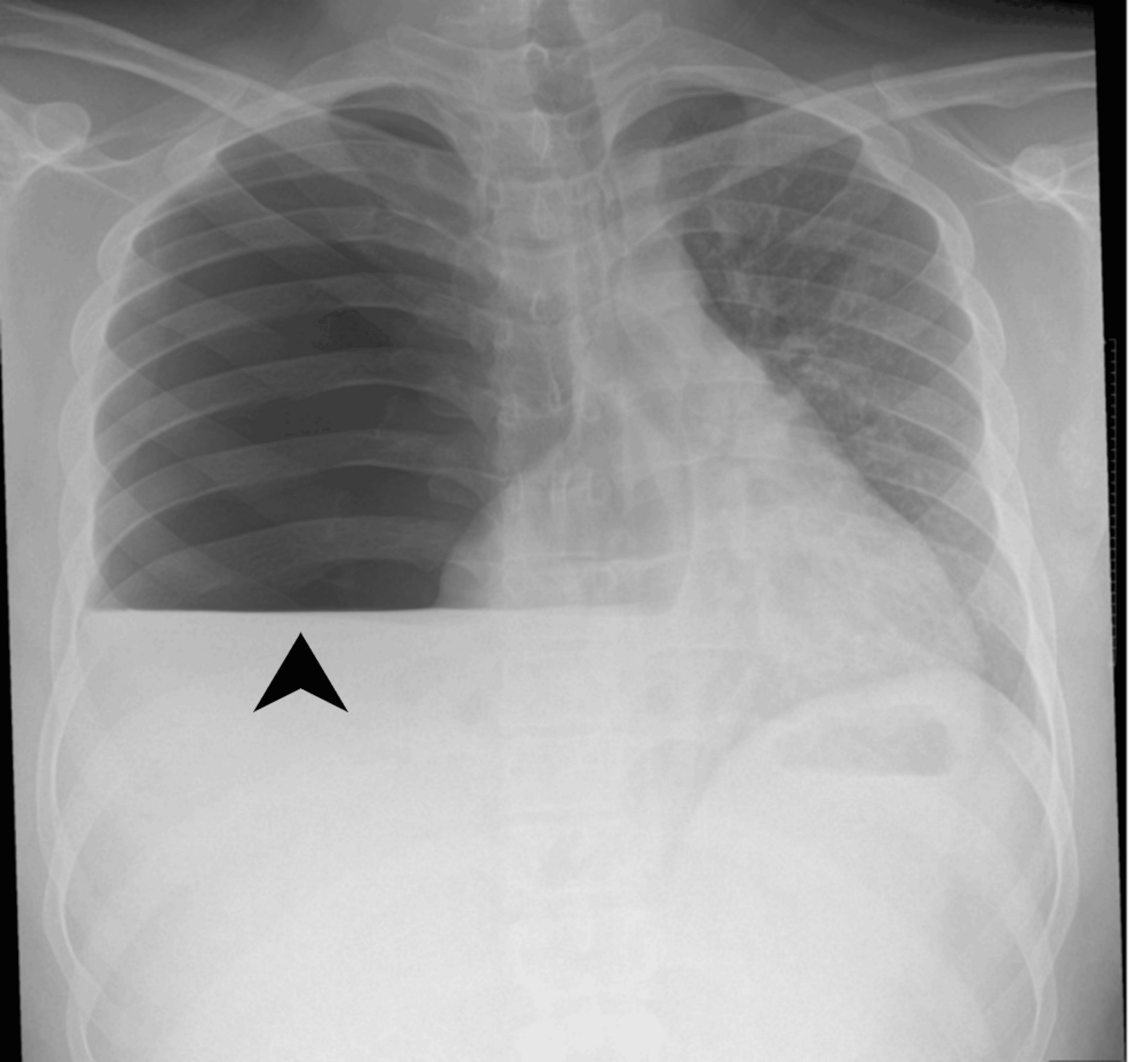
Posteroanterior chest radiograph demonstrating right-sided hydropneumothorax. There is a visible air-fluid level in the right hemithorax, characterized by a horizontal interface suggestive of concurrent pleural air and fluid (arrowhead).

While clinically improving in the IDU, six days later, he developed right-sided pleuritic chest pain and dyspnea. Examination revealed decreased air entry on the right. A chest X-ray demonstrated a right-sided pneumothorax with fluid level and mediastinal shift (Figure 4). An apical chest drain was inserted specifically to address the pneumothorax component, resulting in the evacuation of approximately 1.5 L of air within 2 h.

**Figure 5:**
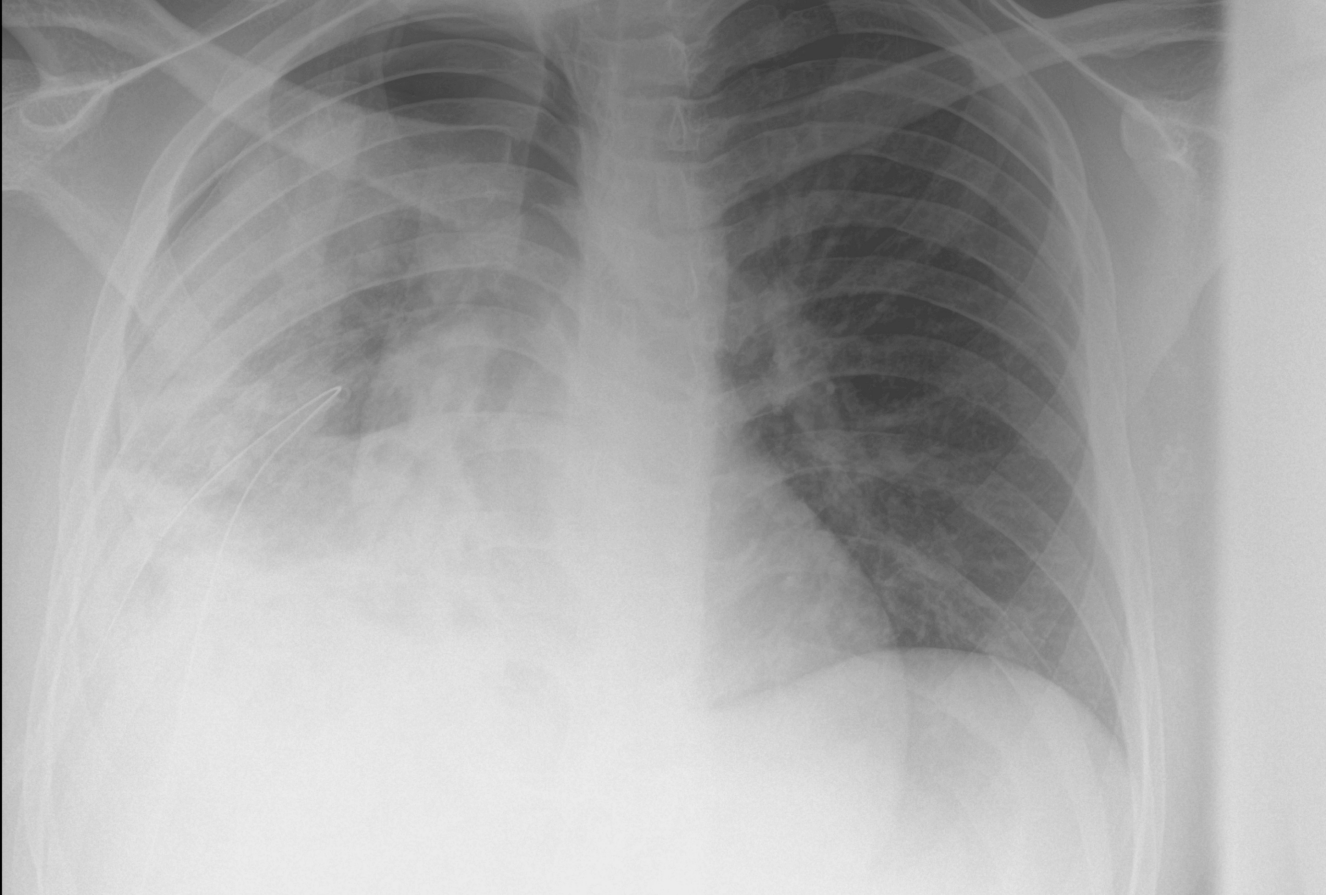
Post-drain chest radiograph showing resolved pneumothorax and residual pleural effusion.

Follow-up chest X-ray showed resolution of the pneumothorax but revealed a persistent pleural effusion (Figure 5). In light of the clinical context and imaging findings, a bronchopleural fistula was suspected at this stage.

The patient was urgently transferred to the respiratory clinical decision unit (CDU), where he was hemodynamically unstable with a respiratory rate of 34/min, oxygen saturation of 92% on 8 L oxygen, heart rate of 148 bpm, blood pressure of 131/76 mmHg, and temperature of 37.9°C. He was transferred to the ICU for 24 h and commenced on continuous positive airway pressure (CPAP). 

**Figure 6:**
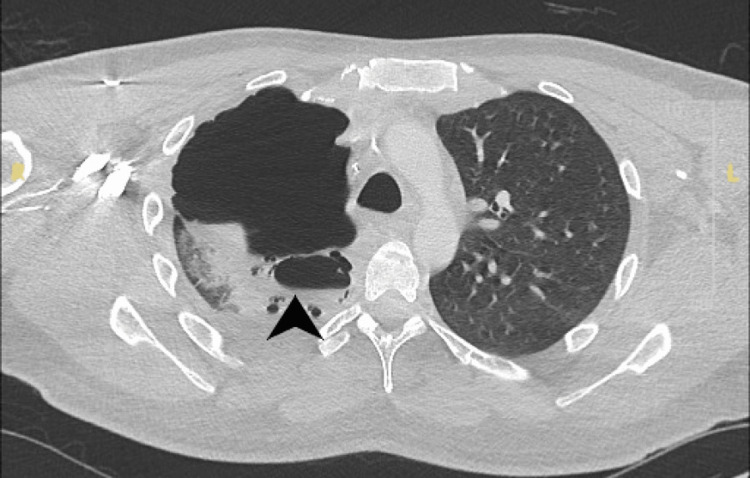
Axial CT thorax demonstrating a complex right-sided multiloculated hydropneumothorax with multiple internal septations. A visible communication between the hydropneumothorax and a cavitating lesion in the right lower lobe (arrowhead) is noted, suggestive of a bronchopleural fistula. CT: computed tomography

**Figure 7:**
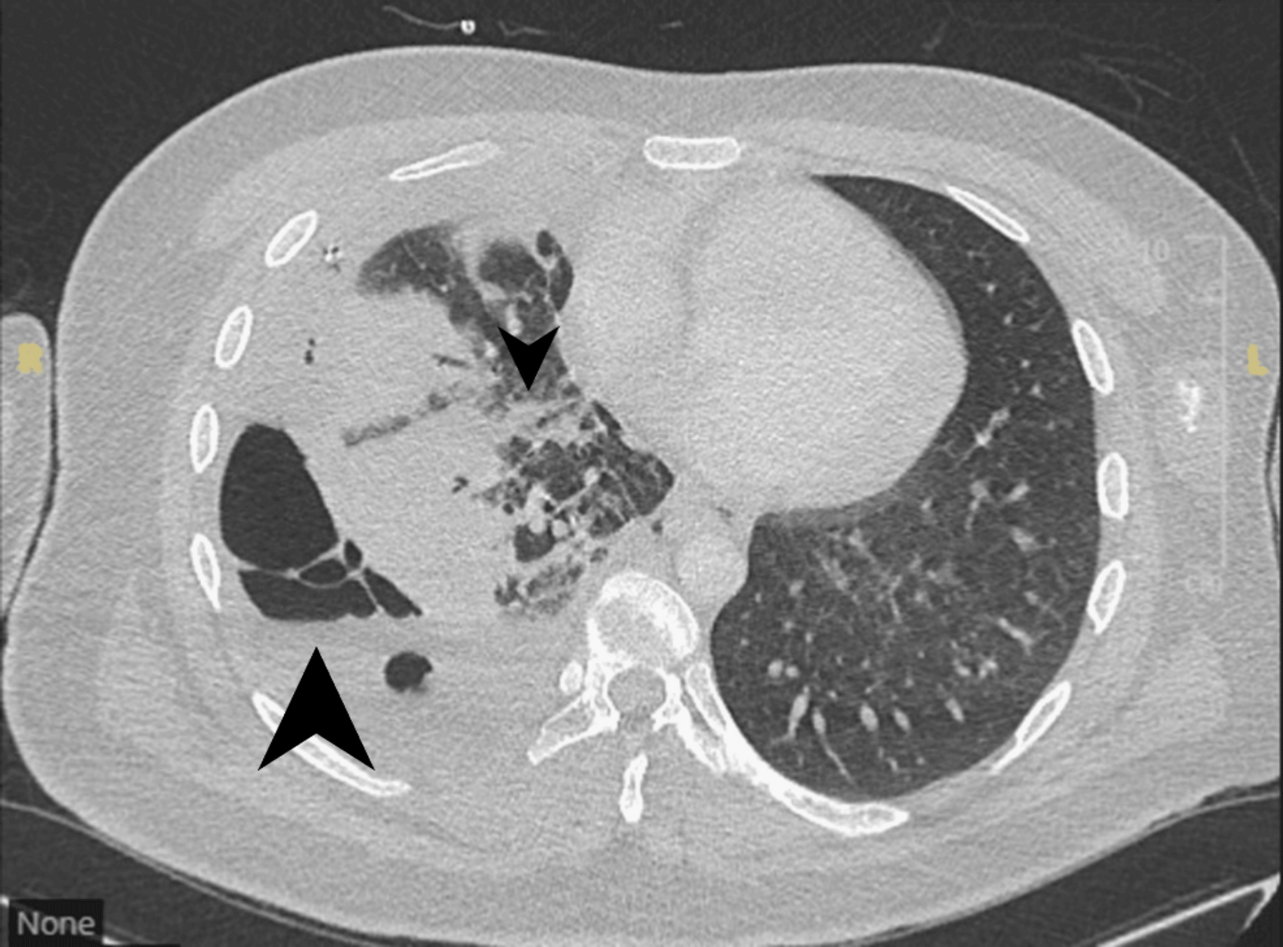
Axial CT thorax at the level of the lower lobes demonstrates a right-sided multiloculated hydropneumothorax with multiple septations. There is clear communication with a cavitating lesion in the right lower lobe (arrowhead), concerning for a bronchopleural fistula. CT: computed tomography

The following day, he stabilized and was transferred to a specialist pleural ward. A follow-up CT thorax revealed a right-sided hydropneumothorax with subcutaneous emphysema (Figures 6, 7). A second chest drain was inserted under CT guidance. The patient continued to experience recurrent hemoptysis, with an estimated volume of approximately 50 mL per episode.

**Figure 8:**
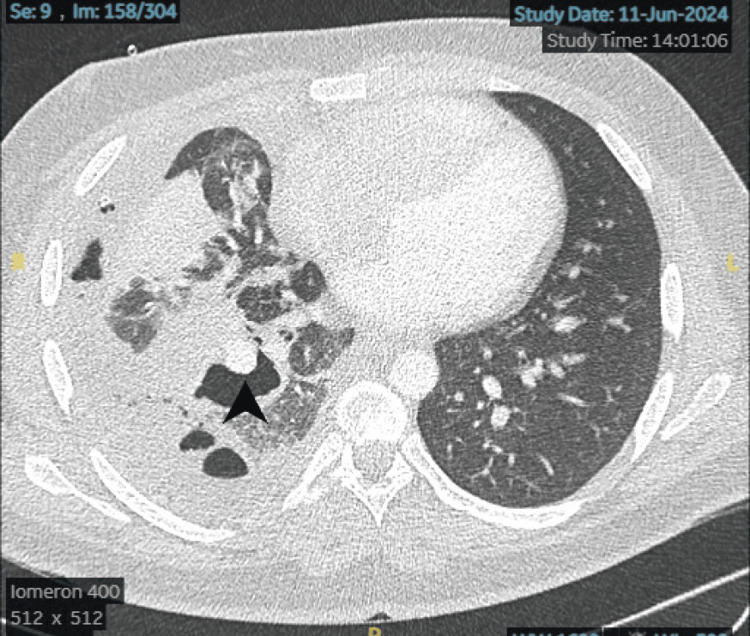
CT aortogram showing a right pulmonary artery aneurysm (arrowhead) located within an infected cavitary lesion in the right lung. The imaging appearance, in the context of active pulmonary tuberculosis, is consistent with a Rasmussen aneurysm. CT: computed tomography

**Figure 9:**
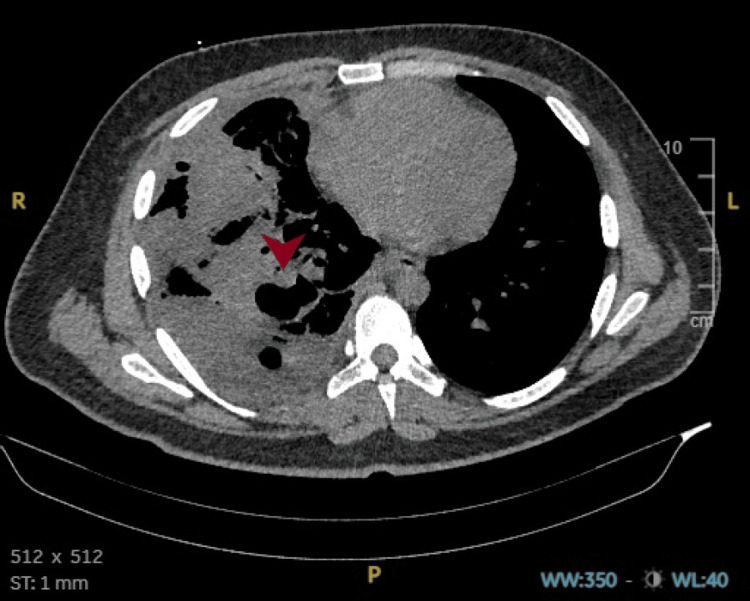
CT aortogram in mediastinal window demonstrating a Rasmussen aneurysm (arrowhead). CT: computed tomography

Ongoing bleeding prompted a CT aorta angiogram, which identified a Rasmussen aneurysm, which was successfully embolized by interventional radiology (Figures 8, 9). This intervention was crucial in controlling the hemoptysis and stabilizing the patient, preventing further vascular complications.

Blood cultures grew Candida species, indicating candidemia, and antifungal therapy with fluconazole and caspofungin was initiated. Following multidisciplinary team (MDT) discussion involving thoracic surgery and interventional radiology, bronchoalveolar lavage (BAL) was performed. BAL cultures grew Klebsiella, Enterobacter, Serratia, Citrobacter (KESC) group coliforms, but no further antibiotics were initiated due to clinical stability. Serial imaging showed TB-consistent changes, including a persistent right-sided hydropneumothorax and newly identified calcified lymph nodes, which had not been observed on prior CT scans.

**Figure 10:**
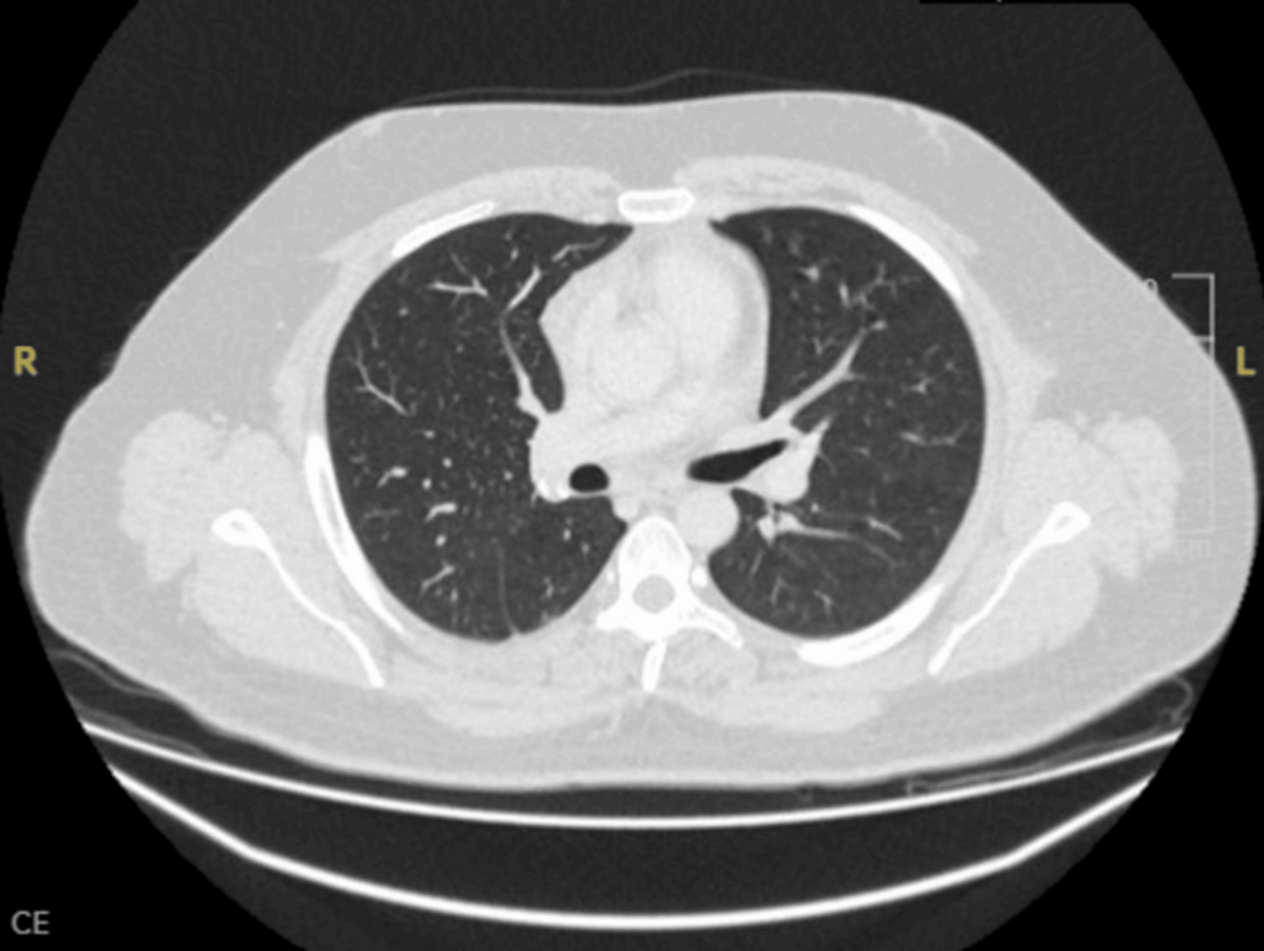
Axial CT thorax showing calcified lymph nodes in the axillary, mediastinal, and upper abdominal regions. The findings are consistent with prior granulomatous infection, such as tuberculosis. There is a noted reduction in the size of the left para-aortic lymph nodes compared to previous imaging. Lung parenchymal changes appear stable. CT: computed tomography

One month into his hospital admission, a final CT scan utilizing streak artefact reduction software excluded pulmonary embolism and confirmed the successful resolution of the Rasmussen aneurysm (Figure 10). The images identified extensive calcification of lymph nodes within the axillary, mediastinal, and upper abdominal areas, reflecting previous granulomatous infection consistent with tuberculosis. Notably, the left para-aortic lymph nodes had decreased in size compared to earlier imaging, indicating a positive treatment response. Lung tissue exhibited stable changes from prior infection without new abnormalities.

**Figure 11:**
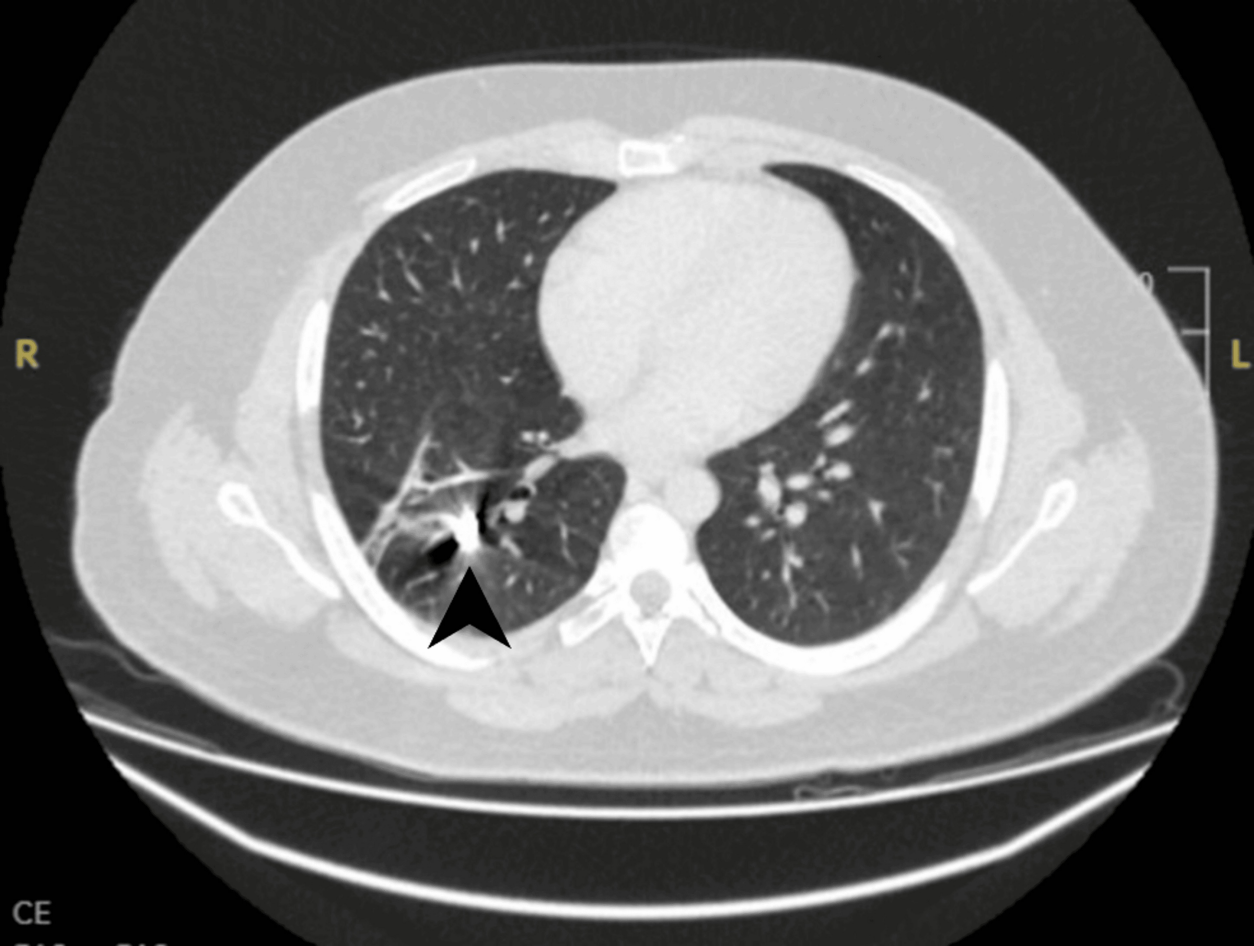
Axial CT thorax showing calcified lymph nodes in the axillary, mediastinal, and upper abdominal regions. At a slightly lower level from the same scan, the coiled Rasmussen aneurysm remained visible in the right lung, with associated metallic artifact from the coil (arrowhead). The findings are consistent with prior granulomatous disease, such as tuberculosis. There is a reduction in the size of the left para-aortic lymph nodes. The lung parenchyma demonstrates stable post-infectious changes. CT: computed tomography

At a slightly lower level from the same scan, the coiled Rasmussen aneurysm remained visible in the right lung, with associated metallic artifact from the coil (arrowhead) (Figure 11). Following these imaging findings, the patient’s antituberculous regimen was simplified to isoniazid and rifampicin. With the removal of all chest drains and stabilization of respiratory status, he was discharged to complete a 16-week continuation phase of therapy.

**Figure 12:**
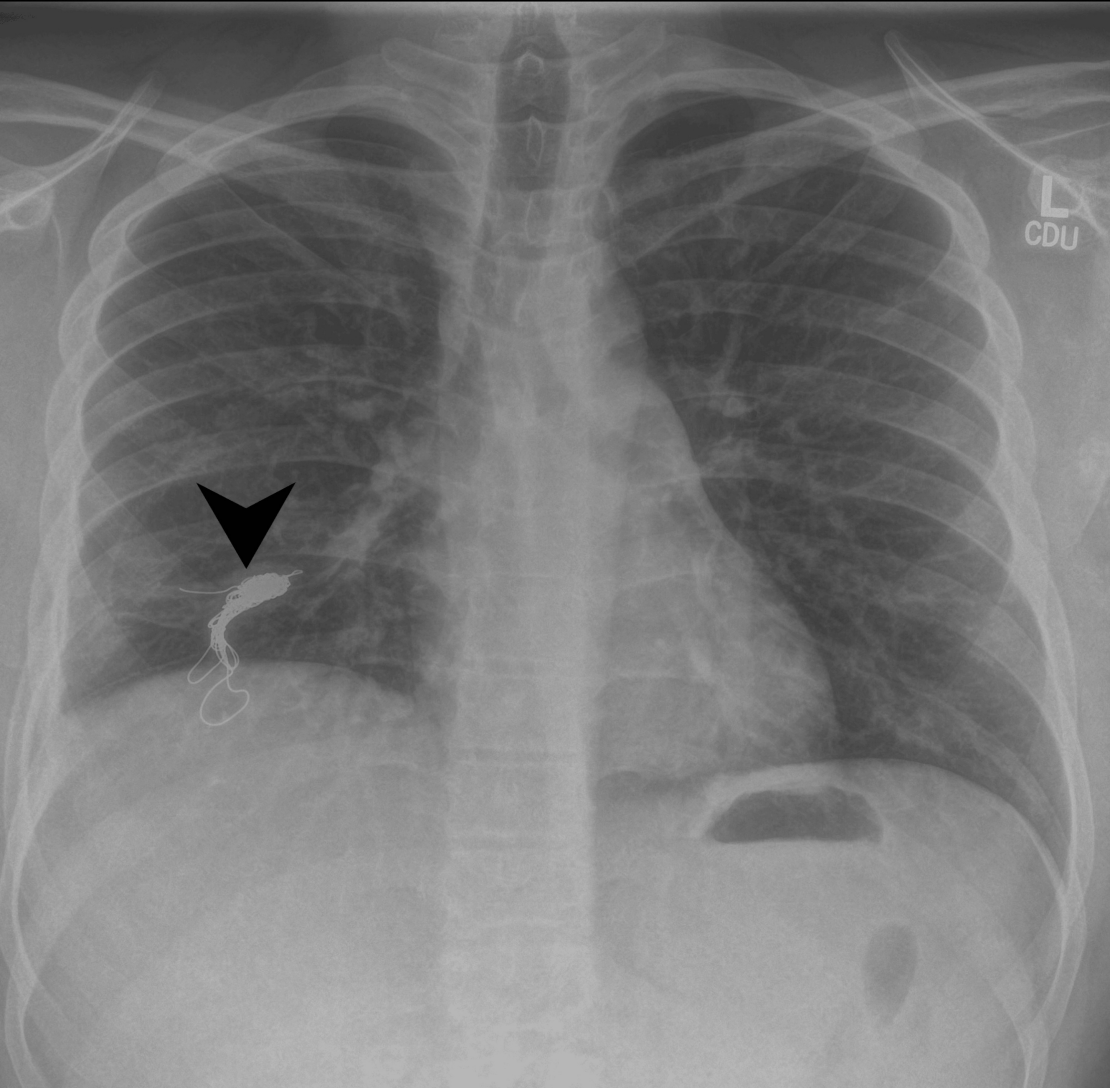
Frontal chest radiograph showing an embolization coil projected over the right lower zone (arrowhead). This finding is consistent with prior treatment of a Rasmussen aneurysm. The lungs and pleural spaces are grossly clear, with no acute abnormalities.

At the five-month follow-up in the pleural clinic, his CT thorax revealed no abnormalities of concern, while the chest X-ray displayed an embolization coil over the right lower lung zone, indicative of previous Rasmussen aneurysm treatment (Figure 12). The lung fields and pleural spaces appeared clear without evidence of acute pathology. Serial imaging had shown gradual resolution of cavitary changes and hydropneumothorax, alongside a reduction in lymph node size. 

He was considered medically stable and subsequently discharged from respiratory follow-up. Table 2 presents the patient’s improved blood test results at this visit, demonstrating resolution of the earlier inflammatory process and clinical recovery. These radiological and biochemical improvements closely mirrored his stepwise clinical progress.

**Table 2:** Laboratory markers at five-month follow-up demonstrating clinical improvement. WCC: white cell count; CRP: C-reactive protein

Test	Result	Reference range
WCC	5.0 × 10⁹/L	4.0-11.0 × 10⁹/L
CRP	13 mg/L	<5 mg/L
Neutrophil count	2.78 × 10⁹/L	1.50-7.50 × 10⁹/L
K	5.0 mmol/L	3.5-5.0 mmol/L
Na	141 mmol/L	135-145 mmol/L

## Discussion

This case presents a rare and diagnostically challenging instance of cavitating pneumonia in a young, immunocompetent male, caused by co-infection with *Staphylococcus aureus* and *Mycobacterium tuberculosis*. While community-acquired pneumonia is a common presentation, cavitation in this context, especially with clinical features such as persistent fever and hemoptysis, should raise concern for atypical or dual infections. Cavitary pneumonia in younger adults is most often linked to aggressive bacterial pathogens such as *Staphylococcus aureus*, particularly post-viral, or in association with aspiration. Although *Staphylococcus aureus* comprises only a small percentage of community-acquired pneumonia (CAP) cases, it has been documented to cause cavitary changes and pneumatoceles in both adults and children [1].

In regions where tuberculosis remains endemic, *Mycobacterium tuberculosis* must remain high on the differential diagnosis when cavitary lesions, lymphadenopathy, or tree-in-bud opacities are noted radiographically. Globally in 2023, an estimated 10.8 million people were diagnosed with TB, a further increase from 10.7 million in 2022, 10.4 million in 2021, and 10.1 million in 2020, underscoring the continued burden and clinical relevance of TB worldwide [3]. In this case, *Mycobacterium tuberculosis* was eventually confirmed as a co-pathogen, contributing not only to persistent pulmonary symptoms but also to a rare and serious vascular complication-Rasmussen aneurysm. First described in the 19th century by Rasmussen, these aneurysms arise from the erosion of the pulmonary arterial wall adjacent to a tuberculous cavity, leading to pseudoaneurysm formation [4]. They can rupture spontaneously within weeks to months of TB symptom onset and are frequently heralded by hemoptysis, which may be massive and life-threatening [4]. In the largest available review of 174 cases, middle artery aneurysms were more likely to rupture (75.4%) than large artery aneurysms (43.5%), and women had a higher rupture rate than men despite lower overall prevalence [2].

The vascular complications in this case were further compounded by pleural involvement, including pneumothorax and empyema. These may result from the rupture of a tuberculous cavity into the pleural space, potentially forming a bronchopleural fistula [5]. This facilitates the entry of caseous material into the pleural cavity, posing a risk of pleural infection and fibrosis with long-term respiratory impairment if not promptly managed [5].

An additional complicating factor was the identification of fungal co-infection, with non-*albicans Candida* species isolated. Fungal co-infections, particularly with Candida, are increasingly recognized in patients with pulmonary tuberculosis [6]. A significant proportion of TB patients, up to 40% in some series, may develop fungal colonisation or infection, with *Candida albicans* being the most common, followed by *Candida tropicalis* (20%) and *Candida glabrata* (20%) [6]. Importantly, non-albicans species often display resistance to first-line antifungal agents, resulting in persistent symptoms even in patients already on antitubercular therapy.

In this case, the cavitary changes may have initially been driven by *Staphylococcus aureus*, creating an environment that facilitated either the unmasking or reactivation of underlying *Mycobacterium tuberculosis *infection. The subsequent identification of Candida species likely reflects the effects of immune stress, prolonged illness, and broad-spectrum antimicrobial exposure. This aligns with findings from a 2017 Iranian study, which reported that tuberculosis accounted for a significant portion of CAP cases and could not be reliably distinguished from other causes based on clinical or radiological features alone, highlighting the diagnostic challenge in endemic areas [7].

The presence of Candida in blood cultures was considered significant and managed with antifungal therapy, whereas the KESC group coliforms isolated from BAL were interpreted as likely colonizers, given the patient’s clinical stability and lack of new radiological deterioration. As such, routine screening for fungal co-infection and antifungal susceptibility testing are recommended, particularly in patients with prolonged or complicated pulmonary courses [5]. Additionally, patients who develop bronchopleural fistulae (BPF) often require long-term follow-up to monitor for complications and ensure adequate healing, as BPF can lead to contamination of the pleural space, resulting in inflammation, empyema, impaired lung expansion, and recurrent pneumonia in the contralateral lung due to aspiration of infected pleural fluid [8].

Despite being young and free of comorbidities, this patient experienced a severe and complex clinical course that required multidisciplinary management involving respiratory medicine, infectious diseases, interventional radiology, microbiology, and thoracic surgery. The case underscores how tuberculosis can lead to life-threatening complications, such as Rasmussen aneurysm, even in immunocompetent individuals. It also highlights the limitations of empiric antibiotic therapy when underlying diagnoses are missed and reinforces the value of early cross-sectional imaging in patients with rapidly progressive pneumonia or atypical radiological findings. Careful outpatient follow-up is equally critical, particularly for patients with risk factors for TB or incomplete clinical resolution. A 2013 prospective study from Indonesia found that over half of pulmonary tuberculosis (PTB) patients continued to experience respiratory symptoms and nearly one-third had moderate-to-severe pulmonary function impairment even six months after treatment, despite achieving microbiological cure [9]. These findings highlight the importance of early diagnosis and the need to assess functional recovery, not just treatment success.

This case further emphasizes the need to reassess patients who do not respond to standard treatment for community-acquired pneumonia. Representation with new or worsening symptoms, especially hemoptysis and hypoxemia, should prompt clinicians to broaden the differential diagnosis to include atypical infections, co-infections, and rare but severe complications like Rasmussen aneurysm. Polymicrobial co-infections, including fungal pathogens, may complicate the clinical picture and delay diagnosis, even in immunocompetent patients. This is particularly important given that fungal co-infections, especially those caused by Candida species, have been linked to substantial morbidity and mortality in TB patients, often presenting with non-specific clinical features that further obscure diagnosis [10].

Therefore, maintaining a high index of suspicion and performing comprehensive microbiological and radiological evaluations are crucial, especially given that disseminated tuberculosis in immunocompetent patients often presents with non-specific symptoms that contribute to diagnostic delays [11]. Timely recognition and escalation of investigations in such cases are essential to avoid diagnostic delays and to initiate targeted, potentially life-saving interventions. This case reinforces the importance of dynamic clinical assessment, flexibility in management strategies, and close interdisciplinary collaboration to optimize patient outcomes in complex respiratory infections.

## Conclusions

This case underscores the importance of considering tuberculosis in patients presenting with cavitating pneumonia, even in the absence of immunosuppression or known TB exposure. The presence of hemoptysis, worsening respiratory status, or failure to respond to treatment should prompt further imaging and microbiological workup. Rasmussen aneurysm, although rare, is a critical complication that requires urgent recognition and intervention. Timely multidisciplinary input and aggressive management can significantly improve outcomes in complex respiratory infections.
